# Odors as cognitive constructs: history of odor classification and **attempts to map odor percepts to physical and chemical parameters**

**DOI:** 10.1093/chemse/bjaf022

**Published:** 2025-07-11

**Authors:** Richard L Doty

**Affiliations:** Smell and Taste Center, Department of Otorhinolaryngology: Head and Neck Surgery, University of Pennsylvania, Philadelphia, PA, 19104 United States

**Keywords:** olfaction, olfactory receptors, history, perceptual objects, sensory coding, chemoreception, physicochemical, odor classification, psychophysics, structure activity

## Abstract

Attempts to map odor percepts to physical and chemical parameters have a long and challenging history. In contrast to color vision, where three classes of photoreceptors respond to the same stimulus property (wavelength), ~400 classes of olfactory receptors are available to respond in a non-linear non-additive fashion to ~5000 different chemical parameters. Theoretically, millions of permutations between structural elements of chemicals and their smells are possible, and some chemicals with different structures have the same odor and vice versa. Importantly, the same odor quality can come from multiple environmental objects and most odors depend upon the synthesis of a complex melody of volatile chemicals that individually can have dissimilar smells. At the individual receptor level, both agonists and antagonists within a mixture can impact receptor function. Hence, it is perhaps not surprising that no universal relationship between odor quality and underlying physical or chemical dimensions analogous to spectral wavelength for vision or air pressure waves for hearing has yet been identified. This review provides a historical account of psychological odor categorization, attempts to map odor percepts to physiochemical parameters, and attendant pitfalls. It concludes that perceived odor qualities may be best viewed as cognitive constructs with attendant variability due to individual experiences, linguistic processes, and biologic factors that do not map well to universal physiochemical dimensions.

## 1. Introduction

Olfaction is largely responsible for the flavor of foods and beverages, mediates multiple aesthetic pleasures, and warns of such hazards as polluted air, fire, leaking natural gas, spoiled food, poor hygiene, and unhealthy environments. Moreover, loss or decrements of smell function can be harbingers for early mortality (Pinto, Wroblewski, Kern, Schumm, and McClintock, 2014; Devanand et al., 2015), as well as numerous debilitating diseases and disorders, including COVID-19 (Moein et al., 2020), Alzheimer’s disease (Murphy, 2019), Parkinson’s disease (Doty, 2017), schizophrenia (Zurlo, Dal Bò, Gentili, and Cecchetto, 2025), and myasthenia gravis (Leon-Sarmiento, Bayona, Bayona-Prieto, Osman, and Doty, 2012). However, despite such importance, this primary sensory modality has been historically denigrated (McGann, 2017), and the paramount goal of identifying universal physical or chemical properties upon which odor quality depends has not been reached.

A number of factors explain why such identification has not occurred, including the complexities of the olfactory system and the synthetic and idiosyncratic nature of odor perception. At the receptor level, nearly 400 different classes of receptor cells—each expressing a different olfactory receptor gene—comprise a population of 6-10 million receptor cells. Each receptor protein is activated by more than one odorant and a single odorant can activate multiple receptors (Malnic, Hirono, Sato, and Buck, 1999; Kajiya et al., 2001; Firestein, 2004; Saito, Chi, Zhuang, Matsunami, and Mainland, 2009). Combinations of activated receptors form distinct combinatorial codes in response to different odorants, although the spatial distribution of such codes is lost in brain regions beyond the olfactory bulb. In most cases, odorants are made up of dozens of chemicals that cannot be individually perceived within their mixtures. At the perceptual level, the majority of odor qualities are based upon learned associations between the percept and the object emitting the odorant (e.g. rose, lemon, coffee), although hedonic attributes, many of which are learned or altered through learning, can also be associated with the percept (e.g. pleasant, nauseous, disgusting).

This review describes how odors have been classified throughout history and elucidates the general lack of consensus regarding such categorization. Various approaches taken to map odor qualities to chemical structures are assessed, including ill-fated attempts to identify odor primaries analogous to color primaries. The failure of both vibrational theories and theories based on physicochemical properties to identify putative vector-related universal stimulus dimensions is noted. The conclusion is reached that perceived odor qualities are best viewed in the final analysis as cognitive constructs with attendant variability due to individual experiences, linguistic processes, and biologic factors—constructs which do not map well to universal physical or physiochemical dimensions and can be viewed as akin to gestalt-like processes seen in facial recognition and language processing. In light of these considerations, it is suggested that it is highly improbable that general theories closely linking odor qualities to sets of such dimensions will be forthcoming.

## History of odor classification

Although rudimentary from the perspective of measurement theory (Stevens, 1951), classification is a key underlying element of odor perception and is fundamental to most theories as to how the olfactory system operates (Harper, Bate-Smith, and Land, 1968). Known attempts to classify odor qualities begin with the early Greeks, followed by seemingly more sophisticated approaches germinating in the 18th century.

### Early Greeks

The early Greeks sought to name odors but found it difficult to classify them into their phenomenology of the world, i.e. being constituents of air, fire, water, and earth. To Plato, most varieties of smells were “distinguished only as painful and pleasant, the one sort irritating and disturbing the whole cavity which is situated between the head and the navel, the other having a soothing influence and restoring this same region to an agreeable and natural condition” (Plato, 1987) (p. 465). This hedonic phenomenology, whose elements are present in many modern odor classification systems, was in accord with that of Democritus and Epicurus, as well as the early Roman atomist Lucretius.

Plato’s student Aristotle divided odors into two main classes: (i) food-related odors present in both air and water and (ii) non-food odors found only in air (e.g. from flowers). Odors within the second class “do not in any degree stimulate animals to feed, nor do they contribute in any way to appetite: their effect, if any, is rather the opposite” (Aristotle, 1987) (p. 681). The pleasantness and unpleasantness of food-related odors were dependent upon whether appetite was sated or not, a concept that has modern parallels (Duclaux, Feisthauer, and Cabanac, 1973; Cabanac and Fantino, 1977).

Theophrastus, Aristotle’s student, reportedly described seven odor qualities, although records documenting them have been lost (Stratton, 1917). He states in “Concerning Odours” that, unlike fire and water, “… earth is the only elementary substance which has a smell, or at least it has one to a greater extent than the others, because it is of a more composite character” (Hort, 1926) (p. 327). He notes that some odors are indistinct and insipid, whereas others, like tastes, have a distinctive character. Odor qualities “appear to correspond to those of tastes, yet they have not in all cases the same names … nor in general are they marked off from one another by such specific differences as are tastes: rather the differences are, one may say, of generic character, some things having a good, some an evil odor.” (p. 327). This sentiment was repeated three decades later by Galen in his *Compendium*: “… some [odors] are said to be pleasant, others irritating. The individual types, however, have no name” (Eastwood, 1981) (p. 69).

### Late Renaissance and 18th century

Odor classification received little subsequent attention until the late Renaissance. Like the Greeks and Romans, many of the classifications at that time were based solely on affective criteria, although towards the end of the 18th century, classifications began to consider chemical structures. In a 1587 thesis at Marburg, Jochim (Johannes) Camerarius the Younger classified odors into heavy, acute, and fragrant categories (Kenneth, 1928). Later, in the mid-1700’s, Albrecht von Haller followed the ancients in employing hedonics, classifying odors into pleasant (e.g. ambrosial), foul (stenches), and intermediate categories, such as roasted coffee (Haller, 1763). Contemporaneously, Carl von Linné (Linneaus) provided an influential 7-category classification of odors in his *Amoenitates Academia*: aromatic, fragrant, ambrosial (musk-like), alliaceous (garlic-like), hircine (goat-like), repulsive, and nauseous (Linnaeus, 1756). Linnaeus indicated that the fragrant and aromatic odors are pleasant (Suaveolentes) and the nauseous and foul odors are unpleasant (Foetidi), and that the odors within the first two classes are “kindly and desirable to our nerves and even to life itself” (Harper et al., 1968). His main reason for classifying odors, however, was to predict the therapeutic effects of a plant from its odor.

A quarter century later, Anne-Charles Lorry developed a 5-category classification system: camphor odors (e.g. labiates, myrtle); narcotic odors (e.g. opium, solanine); etherial odors (e.g. pineapple and other fruits); volatile sour odors (e.g. *Melissa*); and alkaline odors (e.g. onion) (Lorry, 1784). Soon thereafter, Antoine Francois de Fourcroy brought chemistry into his 5-category classification system: extractive or mucous odors, weak-smelling waterless oily odors, volatile oily odors, aromatic acid odors, and odors of sulfurous substances that produce metallic precipitates (de Fourcroy, 1798).

### 19th century

The book that dominated olfactory science throughout much of the 19th century was the 754 page tome of Joseph Hippolyte Cloquet (Cloquet, 1821). Although Cloquet did not develop his own classification system, he noted that Linnaeus and Fourcroy had developed the most comprehensive ones. He stated, however, that von Haller’s use of hedonics in odor classification was too subjective, and pointed out problems of classifying odors into animal, vegetable, and mineral categories. For example, sperm-like odor, which would be classified as animal, is also evident in the flowers of the barberry (*Berberis vulgaris*) and the chestnut (*Castanea vulgaris*).

In 1851, Rudolf Fröhlich functionally differentiated between odorants that primarily stimulate the olfactory nerve (Cranial Nerve I) and those that stimulate the trigeminal nerve (Cranial Nerve V), the latter producing cool, warm, and irritating sensations in the nose, as well as inducing respiratory reflexes such as sneezing (Fröhlich, 1851). He pointed out that some odorants are comprised of multiple chemical components, thereby stimulating both nerves (e.g. tobacco smoke). This differentiation was acknowledged, de facto, by Alexander Bain in 1894, who classified odors into three general groups: first, those that “owe their character to sympathy with the vital organ in alliance with the sense -- namely, the Lungs; secondly, those that appeal to the purely Olfactory sensibility; and, thirdly, those involving an excitation of the nerves of Touch” (p. 165) (Bain, 1894).

Among the most influential odor classification systems of the late 19th century was that of Eugene Rimmel, who introduced the concept of primary odors, a concept described in more detail later in this review. He states on page 10 of his classic book, *The Book of Perfumes* (Rimmel, 1867), that “I have attempted to make a new classification <beyond Linnaeus, von Haller, and Fourcroy>, comprising only pleasant odours, by adopting the principle that, as there are primary colours from which all secondary shades are composed, there are also primary odours with perfect types, and that all other aromas are connected more or less with them.” He classified smells into 18 categories, omitting food odors and unpleasant odors (Table 1). Note that these categories largely reflected raw materials as stimuli rather than pure chemicals.

**Table 1.** Rimmel’s Odor Classification System (1867).

Class	Typical odor	Odors in same class
**Almond**	Bitter Almonds	Laurel, Peach kernels, Mirabane
**Ambergris**	Ambergris	Oak Moss
**Anis**	Aniseed	Carraway, Dill, Coriander, Fennel
**Balsamic**	Vanilla	Benzoin, Storax, Tonka-bean, Heliotrope, Balsams of Peru and Tolu
**Camphor**	Camphor	Patchouli, Rosemary
**Citrine**	Lemon	Bergamot, Orange
**Clove**	Clove	Carnation, Clove-pink
**Fruit**	Pear	Apple, Pineapple, Quince
**Jasmine**	Jasmine	Lily-of-the-Valley
**Lavender**	Lavender	Spike Lavender, Thyme, Marjoram
**Mint**	Peppermint	Spearmint, Balm, Rue, Sage
**Musk**	Musk	Civet
**Orange Flower**	Orange Flower	Acacia, Syringa, Orange-leaves
**Rose**	Rose	Geranium, Sweetbriar, Rosewood, Rhodium,
**Sandal**	Sandalwood	Vetivert, Cedarwood
**Spice**	Cinnamon	Cassia, Nutmerg, Mace, Pimento
**Tuberose**	Tuberose	Lily, Narcissus, Jonquil, Hyacinth
**Violet**	Violet	Orris root, Cassle, Mignonette

In 1895, the father of olfactory psychophysics, Hendrik Zwaardemaker, expanded the Linnaean system of seven descriptive categories to nine, adding Lorry’s class of Ethereal (e.g. the fruity smells of perfumes, beeswax, and ether) and von Haller’s class of Empyreumatic (e.g. smells of roasted coffee, tobacco smoke, and naphthalene) (Zwaardemaker, 1895). His system (Table 2), unlike Rimmel’s, sought to include specific known chemicals, when available, rather than raw materials or complex mixtures of essential oils. He also sought to omit chemicals that primarily produced taste sensations and tactile nasal responses related to the trigeminal nerve, although the latter was clearly not achieved in relation to what is known today (Doty, Brugger, et al., 1978). Additionally, he developed subclasses of some of his nine basic categories. It is noteworthy that in his classification scheme, some odorants could fall into more than one category (e.g. pyridine).

**Table 2.** Zwaardemaker’s Odor Classification System (1867).

Class 1: Ethereal	Examples: acetone; chloroform; ethyl ether; ethyl acetate; pentyl acetate; ethyl butyrate, methyl butyrate; all fruit odors
**Class 2: Aromatic**	
** Subclass**	**Examples:**
* Camphoraceous*	camphor, borneol, pinene
* Spicy*	eugenol
* Aniseed*	anethole, menthol, methyl salicylate, safrole, thymol
* Citrus*	citral, geraniol, linaloöl
* Almond*	benzaldehyde, nitrobenzene, benzonitrile
**Class 3: Floral and Balsamic**	
** Subclass**	**Examples:**
* Perfumes of flowers*	ethyl anthranilate, phenyl ethyl alcohol, terpineol
* Lily type*	ionone, irone, piperonal
* Vanilla*	vanillin, coumarin, anisaldehyde
**Class 4: Ambrosiac**	**Examples:** amber, muscone, artificial musk
**Class 5: Alliaceous**	
** Subclass**	**Examples:**
* Garlic*	acetylene, hydrogen sulfide, mercaptans (thiols),
* Cacodylie*	cacodyl, trimethylamine
* Bromine (type)*	bromine, iodine
**Class 6: Empyreumatic**	**Examples:** acrolein, amyl alcohols and homologues, anthracene, benzene, cresol, guaiacol, naphthalene, petroleum, phenol, pyridine, toluene, toluidine, xylene
**Class 7: Caprylic**	**Examples:** “acide capronique” and homologues; pentamethylenediamine (cadaverine)
**Class 8: Repulsive**	**Examples:** alkaloids, pyridine, quinolone, opium
**Class 9: Foetid**	**Examples:** indole, skatole

Zwaardemaker viewed odor classification at the end of the 19th century akin to that of color classification before Newton’s spectral analyses of color. At that time, colors were identified and named in terms of “universal images,” much like most odors are named today based upon the physical objects from which they arise (Harper et al., 1968). As noted by Hermann von Helmholtz, the color red comes from the Sanskrit word for blood, blue from Greek and Latin references to the sea and sky, respectively (*πορϕύρϵος* and *coeruleus*, from *coelum*), and green from the Greek word for leek-green vegetation (*πράσινος*) (Helmholtz, 1860). To Zwaardemaker, an ideal classification system would be one that could accommodate all existing odors as well as new ones developed in the future.

### 20th and 21st centuries

The 20th and 21st centuries heralded the beginning of experimentation in the development of odorant classes. In 1915, the Swiss psychologist P. Kennel chose 39 odorants from 8 of Zwaademaker’s classes (Kennel, 1915). He had 35 well-educated subjects attempt to divide their odors into groups. Most were mixtures (e.g. coffee, mustard), but some were pure compounds such as camphor, ether, and iodoform. He found that while a few odorants could be reliably grouped together in one or two of Zwaardmaker’s categories, most could not, and a number were assigned to more than a single group. Other psychologists subsequently pointed out these and other problems with Zwaardmaker’s classification system (Henning, 1916; Titchener, 1928; Hazzard, 1930).

**Fig. 1.**
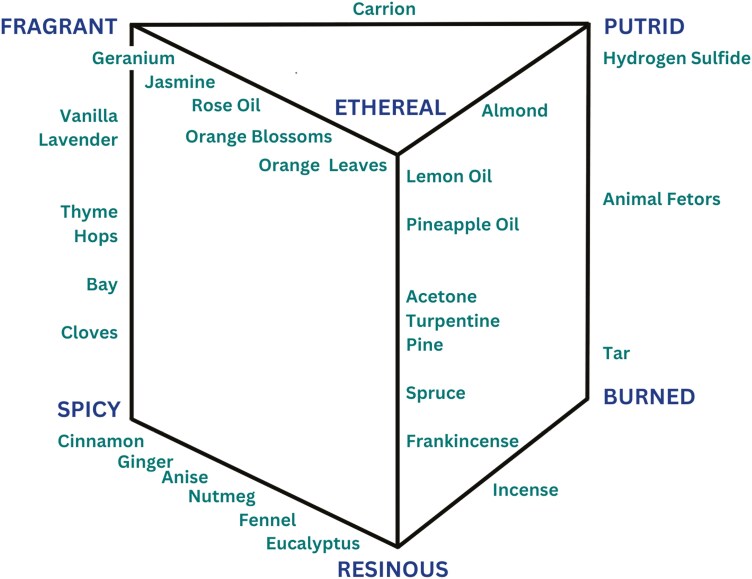
Henning’s “odor prism” (1916). Odors were placed relative to six standards (corners of prism) on the faces of the prism. Examples of odors located along the edges of the prism are indicated. Other odors were located on the three faces of the prism.

An important conceptual advance in odor classification of this period was the idea that relationships of odors to one another could be placed into three-dimensional space, a concept that has modern analogs (e.g. Schiffman, 1974). Hans Henning pioneered this novel approach by having subjects determine where odors should be placed relative to the surfaces of a three-dimensional “odor prism” whose corners were defined by exemplars of fragrant, spicy, resinous, ethereal, burned, and putrid (Fig. 1) (Henning, 1916). The two triangular faces were equilateral; squares made up the connecting faces. Nearly 3000 judgements were made by Henning and his students, and over 400 odors were classified into his system, including pure chemicals and natural products. He stressed, however, that a mixture of two odors does not give rise to an intervening odor on the prism. Importantly, he recognized an inherent hedonic element to his classification system stating, “A secant plane divides the olfactory prism into a pleasant half and an unpleasant half. On the pleasant side lie the first four classes of odors (spicy, flowery, fruity and resinous), on the unpleasant the two last classes (putid and burnt). Indifferent odors would lie along the section.” (p. 181–182)

Despite its creative representation of the potential multidimensionality of odors, Henning’s odor prism fell out of favor due to failures of replication (Dimmick, 1922; Macdonald, 1922; Findley, 1924) and confounding by trigeminal sensations (Bentley, 1926). For example, Agnes Elme Findley noted, in her attempt at replication, that, “In general, the differences <in responses among the categories> are closer to ‘chance than to perfect consistency” (Findley, 1924) (p. 439).

The most important methodological advance in odor classification after Henning was a numbering system developed in 1927 by the chemical engineer Ernest C. Crocker and industrial chemist Lloyd F. Henderson (Crocker and Henderson, 1927). Specifically, they sought to quantify the extent to which odorants exhibited basic odor qualities of Fragrant, Acid, Burnt, and Caprylic. Employing exemplars of each of these four odor qualities, subjects used an 8-point scale to rate the degree to which a given odorant represented these qualities. For example, vanillin was codified as 7122, reflecting its predominantly fragrant quality, whereas acetic acid received the code of 3803, reflecting its acidic nature. Extensive listings of both natural and synthetic aromatic chemicals were developed using this system, and a set of 32 odorant standards for their system was made commercially available in 1949 (Crocker and Dillon, 1949).

While Crocker and Henderson’s approach takes into account the multidimensional qualities of individual odors, its use was ultimately abandoned due to its complexities, lack of reliability, propensity for odor fatigue, and confusions among subjects in making the ratings (Ross and Harriman, 1949; Sagarin, 1950; H. G. Schutz, 1964). Moreover, the basis for assuming that the four qualities represented the entire odor space is not clear, despite their demonstration of different patterns of changes in perceived intensity across carbon chain lengths of their exemplars of the four qualities (Crocker and Henderson, 1927).

In 1985, Andrew Dravnieks published the American Society for Testing and Materials (ASTM) Atlas of Odor Character Profiles (Dravnieks, 1985). This Atlas was based on the semantic profiling of 180 odorants. A list of 146 odor descriptors, selected from an initial list of 800, was provided to over 100 panel members from different organizations who determined, for every odorant, the applicability of each descriptor on a scale from 0 to 5. The stimuli consisted of odor-impregnated balsa wood chips enclosed in small individually sealed aluminum packets that were easily opened to release odorant. Two statistical measures were obtained: (a) the percent of subjects who used a particular descriptor for a given stimulus and (b) a score based on the numerical ratings. For vanillin, for example, the highest percentages of responses were “fragrant” (40.91%), “chocolate” (48.32%), “vanilla” (67.11%), “malty” (32.89%), and sweet (65.77%). Although not generally subjected to reliability analysis, high test–retest reliability was reported for responses of over 100 trained panelists to 10 odorants evaluated by this method (Dravnieks, 1982).

More recently, the ASTM and other large databases of odor descriptors (e.g. Arctander, 1969; Boelens and Haring, 1981; Thiboud, 1991; Leffingwell, 2001) have been subjected to factor or principal component analyses, multidimensional scaling (MDS), and other statistical procedures in attempts to characterize odor descriptor space in low-dimensional perceptual maps (see, e.g. Mamlouk and Martinetz, 2004; Koulakov, Kolterman, Enikolopov, and Rinberg, 2011; Lee et al., 2023). In some cases, two- and three-dimensional “maps” have been reported to be useful to perfumers by illuminating consumer preferences, describing complex odorant mixtures, training sensory panels, and improving communication among perfumers, retailers, and customers (Zarzo and Stanton, 2009).

That being said, a number of authors have pointed out that odor classification systems based on odor descriptors have multiple shortcomings, resulting in significant variability that limits the degree to which such descriptors represent odor space and can be meaningfully employed in structure–activity relationships. Among the major shortcomings that have been noted are as follows:

- Most odor names are derived directly (e.g. coffee) or indirectly (e.g. fruit-like) from their source. In 1879, Thomas H. Huxley pointed out that this is metaphysically unsound, stating, “… it is as absurd to suppose that muskiness is a quality inherent in one plant, as it would be to imagine that pain is a quality inherent in another because we feel pain when a thorn picks the finger. That which is true of muskiness is true of every other odour; lavender-smell, clove-smell, garlic-smell, are, like ‘muskiness’, names of states of consciousness, and have no existence except as such.” (Huxley, 1879) (pp. 600–601).
- In accord with Huxley’s observation, Holley and MacLeod (1977) pointed out the paradox that “Only the source of an odor is truly apprehended as an entity, to the point that we are unable to give a name to the latter save via the former. We lack the rigor of language required for more precise description and are forced to fall back on metaphors.” (p. 720).
- Odor classes are language and culturally dependent (Pangborn, Guinard, and Davis, 1988; Song and Bell, 1998; Fornazieri et al., 2013; Kaeppler and Mueller, 2013; Keller and Vosshall, 2016). Just as cultures living in northern climes have names for types of snow lacking in English (Magga, 2006), some have many more odor names than others (Majid, 2021). Familiarity with odorants impacts how they are assigned to verbal descriptors (Keller and Vosshall, 2016).
- Employing many descriptors or standards to which to compare odors is impractical, as thousands are available. Even for a subclass of odors, such as those from wine, dozens of descriptors exist (e.g. a widely employed aroma wheel for describing wine includes 87 descriptors; Noble, 1987).
- Subtle differences among odors require different classifications, thereby exacerbating this issue. As pointed out by Zwaardemaker (1925), “If we choose to give the same name to several similar odors, it could often happen that the evidence of our designation eludes other people … and one may question the legitimacy of our identification.” (p. 179)
- Determining the number of classes is highly subjective and large differences exist among individuals in assigning odorants to extant odorant classes (Berglund, Berglund, Engen, and Ekman, 1973). The latter reflects, in part, marked differences among individuals in their receptor gene repertoires (Keller, Zhuang, Chi, Vosshall, and Matsunami, 2007; Hoover, 2010; Olender et al., 2012) and is evident in ethnic and geographically distinct populations (Menashe, Man, Lancet, and Gilad, 2003).
- Perceived odor quality can vary with odorant concentration (Demole, Enggist, and Ohloff, 1982; deMoncrieff, 1967; Theimer, Yoshida, and Klaiber, 1977; Gross-Isseroff and Lancet, 1988; Wijk and Cain, 1994), as can odor pleasantness (Doty, 1975), confounding class assignments.
- Odor qualities are labile, being influenced by such factors as subject age (Doty and Kamath, 2014), subject gender (Doty and Cameron, 2009; Sorokowski et al., 2019), relatively common diseases (Liu, Zong, Doty, and Sun, 2016; Doty, 2019), and whether the odorant is sniffed (orthonasal route) or enters the back of the nasal cavity (nasopharynx) from the oral cavity, as occurs during eating (Hannum, Stegman, Fryer, and Simons, 2018; Pellegrino, Horberg, Olofsson, and Luckett, 2021). Interestingly, the ability to perceive some compounds reliably appears only after repeated testing in some persons (Van Toller, Kirk-Smith, Wood, Lombard, and Dodd, 1983; Wysocki, Dorries, and Beauchamp, 1989; Dalton, Doolittle, and Breslin, 2002).
- As indicated by Harper et al. (1968), context and need are major drivers for the development of a classification system: “In the case of food it is primarily the recognition of what is eatable and desirable and what is not, and classification can simply be achieved in terms of ‘desirable’ and ‘undesirable’” (p. 105).

The difficulties in classification have been appreciated throughout the 20th century. In 1927, the American chemist Marston T. Bogert opined, “An exact and impersonal scientific classification of odors, comparable to that available for colors, seems clearly unattainable in the light of our present knowledge. The best that can be done is a more or less superficial grouping into types or classes which to the observer seem to bear a sort of family resemblance to one another, and we therefore speak of (for example) lily type, rose type, musk type, of odor, but the boundaries of these groups are both vague and variable” (Bogert, 1927) (p. 5). This sentiment was echoed a quarter of a century later by Edward Sagarin (Sagarin, 1950), as well as by Sherman Ross and Arthur Harriman. The latter two investigators concluded, “No physical, chemical nor psychological approach, nor any combination of them, provides a generally useful classification of odors by which even trained observers may reliably describe or classify these qualities” (Ross and Harriman, 1949) (p. 399). As noted later in this review, this sentiment remains today despite advances in computer technology, identification of the odorant receptors, and improvement in olfactory measurement procedures (Kaeppler and Mueller, 2013).

A number of 20th century investigators sought to avert the variability in semantic tasks by defining odorant classes using similarity estimates (also termed proximity analyses) and other non-semantic procedures. For example, in one paradigm, sets of odorants are smelled, usually two at a time, and their similarity is noted on a rating scale. The ratings can be subjected to factor analysis and MDS, the latter of which places the odorants in geometric space relative to their similarities to one another (Jaworska and Chupetlovska-Anastasova, 2009). As Amos Tversky pointed out, “It <similarity> serves as an organizing principle by which individuals explain and classify objects, form concepts, and make generalizations. Indeed, the concept of similarity is ubiquitous in psychological theory” (Tversky, 1977) (p. 327).

Nonetheless, like semantic odor descriptors, large individual differences have been found in odor similarity ratings and the resulting scaling structures, leading some to conclude that group data are not representative of individual data (Gregson, 1972; Berglund et al., 1973). Moreover, subjects may emphasize different perceptual attributes for different sets of odorants (Engen, 1973), and it is questionable whether putative non-semantic paradigms are totally free of semantic influences. Thus, direct comparisons of odors may require a mental search through odor names, involving both perceptual and verbal processes (Kaeppler and Mueller, 2013). Odor discriminability, which is often assumed to be independent of semantic processing, is strongly correlated with the ability to identify odors (de Wijk and Cain, 1994). A key element of factor analytic, cluster analyses, and similar statistical paradigms is to ultimately name the resultant factors or classes, a process that involves semantic classification if one seeks to understand odor perception, per se.

In most cases, hedonics has appeared as a major dimension when similarity or other non-semantic estimates are subjected to such analyses (e.g.Beebe-Center, 1929; Davis, 1979; Haddad et al., 2008; Hsü, 1946; Khan et al., 2007; Schiffman, Robinson, and Erickson, 1977; Schiffman, 1974; Schutz, Vely, and Iden, 1961; Stephenson, 1936; Yoshida, 1964a). Even Zwaardemaker’s odorant classes can be differentiated on the basis of hedonics (Eysenck, 1944). Thus, in accord with the perspective of the early Greeks, hedonics appears to be a common element of the olfactory experience. Nevertheless, as pointed out by Birgitta Berglund and Anders Höglund (Berglund and Höglund, 2012), “Pleasantness and unpleasantness are not inherent odour-specific concepts; rather they are hedonic or aesthetic evaluations freely applicable to any of our sensory perceptions (colours, music, touch, flavour)” (p. 13).

That being said, a case can be made that olfaction’s raison d’êntre is evaluative rather than descriptive (Castro and Seeley, 2014; Keller, 2016). The hedonic aspect of odor perception appears to trump that of the other sensory systems (Skrzypulec, 2023) and likely reflects, in part, the direct projections of a number of olfactory bulb neurons to the amygdala in an apparent labelled line fashion (Wilson and Sullivan, 2011). From this perspective, the olfactory system’s evolutionary mantra is to sense healthy and unhealthy, or positive and negative, aspects of the environment—aspects to which odor quality identification and classification would lend support but need not be essential. As pointed out by Stevenson (2010) and in accord with the evaluative function hypothesis, key olfactory-related functions, such as ingestion, avoiding environmental hazards, and social interactions, need not require the identification or naming of odors. Like odor qualities, however, odor hedonics is variable and labile (Bontempi, Brand, and Jacquot, 2024), being influenced by learning or experience (Ayabe-Kanamura, Saito, Distel, Martinez-Gomez, and Hudson, 1998; W.S. Cain, 1979), odor intensity (Henion, 1971; Doty, Orndorff, Leyden, and Kligman, 1978), physiological state (Cabanac, 1971), and subject expectations (Slosson, 1899; Engen, 1972; Dalton, 1996). Regarding the latter, odors are often rated as more unpleasant when subjects are told they are potentially harmful than when told they are safe (Zucco, Militello, and Doty, 2008), unmasking top-down influences.

### Brief History of Physical and Chemical Theories of Odor Qualities

Scientific attempts to identify physical and chemical processes upon which odor quality classification depends began in earnest with 19th century advances in chemistry, many occurring in light of Mendeleev’s development of the periodic table in 1869. Dozens of papers explicating diverse theories of smell subsequently emerged, including ones based upon antibodies, enzymes, and mucus adsorption boundaries (for reviews, see von Skramlik, 1926; McCord and Witheridge, 1949; Airkem, 1952; Jones and Jones, 1953; Moncrieff, 1967; Harper et al., 1968; McCartney, 1968; Davies, 1971; Cain, 1978). Those involving molecular vibrations and general molecular structures, including molecular sizes and volumes, are the most prevalent today, but suffer from many shortcomings, as briefly reviewed below.

### Molecular vibration theories

Because wave energy was found responsible for vision and hearing in the 19th century, it is not surprising that many scientists of that era believed that odor qualities and their classifications were dependent upon radiations or molecular vibrations. This provided an underlying physical dimension analogous to spectral wavelength for vision or air pressure waves for hearing. Prior to the discovery of the olfactory receptor genes (Buck and Axel, 1991) and evidence that the expressed olfactory receptors respond to odorant ligands (Krautwurst, Yau, and Reed, 1998; Zhao et al., 1998; Touhara et al., 1999), vibration theories were, of necessity, relatively general and largely phenomenological. This is less true in later reincarnations of vibration theories, which have focused on ligand/receptor interactions, although the molecular prerequisites for establishing their validity, such as the crystalline structure of most odorant receptors, remain enigmatic.

R.C. Rutherford (1882) indicated that he had first noted in 1863 that since a grain of musk can “perfume for several years a chamber twelve feet square without sustaining any sensible diminution of its volume or weight” (p. 84), the odorous property itself is

… purely a specific variety of motion as the undulations of the luminiferous ether. That this *must* be the explanation of the action of the odor-generating force for a part of its route to the human sensorium seems to be incontrovertible, for it is hardly conceivable that the material particles should actually penetrate the <Schneiderian> membrane and force their way, as moving bodies, through the pulpy tissue of the nerves to the seat of sensation; but that through that portion of their career, at least, their power is propagated by wave-like motions analogous to those of heat and sound.” (p. 84)

Among the most prominent 19th century vibration theories were those of George William Septimus Piesse (Piesse, 1867) and William Ogle (Ogle, 1870). Piesse placed various odors on a musical scale (e.g. camphor vibrated one octave above middle C), whereas Ogle proposed that odorants were absorbed into pigment within the upper recesses of the nose. The vibrations of the molecules were then converted into vibrations of heat, which led to the perceived odor. Sir William Ramsay, who discovered the noble gases for which he received the Nobel Prize in Chemistry in 1904, explored the notion of molecular vibration in a manner similar to that of Piesse but different from the heat theory of Ogle (Ramsay, 1882) (p. 188):

There is a probability that our sense of smell is excited by vibrations of a lower period than those which give rise to the sense of light or heat. These vibrations are conveyed by gaseous molecules to the surface network of nerves in the nasal cavity. The difference of smells is caused by the rate and by the nature of such vibrations, just as difference in tone of musical sounds depends on the rate and on the nature of the vibration, the nature being influenced by the number and pitch of the harmonics.

Five years later, John Berry Haycraft provided an interesting ontogenetic and comparative rationale for the molecular vibration theory (Haycraft, 1887):

The end-organs of the special senses are all built upon the same type. The history of their development from simple ectodermic cells suggests that similar agencies have been at work to produce them. Both sapid and odorous substances, and indeed all gaseous and liquid molecules, are now known to be in constant vibration, and this vibration is characteristic of the substance examined. (p. 207)

Numerous odor vibration theories appeared in the 1900s based on infrared and Raman spectrometry. Infrared spectroscopy measures the amount of infrared light absorbed when passed through a chemical sample, providing a unique “fingerprint” of the chemical’s molecular vibrations (Stuart, 2004). In Raman spectrometry, a monochromatic light is focused on a chemical sample, altering its molecular activity (Larkin, 2017). The difference between the wavelength of the focused light and that of the sample’s reflected wavelength, termed the Raman Shift, provides an index of the molecule’s vibrational frequency.

In 1919, Albert Heyninx proposed that odorous chemicals have ultra-violet vibrations with wavelengths ranging from 350 nm to 200 nm (Heyninx, 1919). He posited that seven fundamental classes or categories of odors, namely sharp, rotten, fetid, burnt, spicy, vanilla, and ethereal, fell within these parameters. After being absorbed into the olfactory mucus, these vibrations were somehow transmitted to olfactory receptors and intensified by pigment within the receptor region.

In 1938, Malcolm Dyson hypothesized that all odorous substances have a Raman shift spatial frequency between 1400 cm^−1^ and 3500 cm^−1^, and that similarly smelling chemicals had similar Raman shifts (Dyson, 1938). In contrast, Robert Hamilton Wright concluded a decade and a half later that the spatial frequencies of molecular vibrations within the far infrared spectrum were those that were involved in receptor activation (i.e. 85 cm^−1^ to 340 cm^−1^) (Wright, 1954b). His rationale was that only a few odorant molecules had vibration frequencies in the range noted by Dyson and that this region largely reflected chemical properties of odorants (Wright, 1954a, 1977; Wright, Hughes, and Hendrix, 1967). In one study, he presented data that he claimed demonstrated an association between odorant vibration frequencies and neural firing frequencies of rat olfactory bulb output neurons (Wright, 1968). Around the same time, Rudolf Randebrock proposed a vibration theory that assumed that peptide chains within α-helices are vibrating (Randebrock, 1968). An odorant molecule attaches itself to one end of the peptide to modulate the vibration, which is then somehow transferred to a nerve cell. No empirical data were collected.

More recently, Luca Turin developed a vibration theory with a specific focus on G protein-coupled receptors (GPCRs)—receptors known today to be responsible for binding odorants introduced into the nasal cavity (Turin, 1996). Briefly, he argues that such receptors are sensing molecular vibrations via inelastic electron tunneling, i.e. when odorant electrons tunnel to the receptor site and lose part of their energy by exciting vibrations within the receptors. Details of his theory are provided elsewhere (Brookes, Horsfield, and Stoneham, 2012; Gane et al., 2013; Block, Jang, Matsunami, Batista, and Zhuang, 2015; Turin, Gane, Georganakis, Maniati, and Skoulakis, 2015; Drimyli, Gaitanidis, Maniati, Turin, and Skoulakis, 2016; Maniati, Haralambous, Turin, and Skoulakis, 2017; Block, 2018.

### Challenges to vibration theories of odor qualities and classifications

The plausibility of vibration theories in explaining and cataloguing odor qualities has been questioned for many years on numerous grounds. The following observations are in opposition to elements of such theories:

- The early notion of Ogle and others that radiation or heat from vibrating molecules induces olfactory responses was dispelled in a simple experiment by the physiologist David Ottoson. He eliminated summated electrical potentials generated by odorants in the frog olfactory epithelium by placing a thin membrane over the receptor cell surface that transmits only light and heat (Ottoson, 1956).
- Rutherford’s claim that musk did not lose weight after long periods of air exposure, thereby implicating vibrations, was disproven by Charles Bazzoni using a flexure microbalance (Bazzoni, 1915), an ultrasensitive balance comprised of a quartz fiber whose flex in response to vapor pressure is detected using a calibrated microscope.
- Pigment, which was presumed by Ogle and others to be essential in transferring molecular vibrations into neural activity, is lacking in olfactory receptor cells of rodents and other mammals that have been evaluated, including those with an excellent sense of smell (Moulton, 1960). When present, it is found in non-neural cells within the olfactory epithelium, including the main source of mucus in the olfactory receptor region— Bowman’s glands (Moulton, 1971).
- Some odorants with identical infrared spectra do not smell the same (e.g. menthol and carvone), negating the notion that their odor quality is caused by vibrations in the infrared region (Sharma et al., 2019).
- Similarly, odorants with very different odors can have essentially the same ultra-violet absorption bands (e.g. cinnamic aldehyde and iodoform)(McKenzie, 1926).
- Optical isomers—molecules with identical chemical formulae but which cannot be superimposed upon one another—can have identical Raman frequencies but different smells (e.g. d- and l-carvone smell of caraway and spearmint, respectively), showing that vibrational frequencies alone cannot explain such differences in odor quality (Engen, 1982).
- Accordingly, odorants with the same smell can have completely different Raman spectra (e.g. hydrogen sulfide and deuterium sulfide) (Moncrieff, 1967).
- Wright’s curves denoting correlations between vibration frequencies and olfactory bulb afferent neural frequencies can be replicated by employing ordered sets of random numbers (Davies, 1971).

To address the evidence that isomers with the same vibrational frequencies can have different smells, Wright (1964) argued that the frequency pattern of the isomers may diverge at the moment of interaction with the receptor. However, evidence for this point is lacking. Moreover, subsequent studies have altered the structure of odorants and employed receptor technology in ways that further throw into question the validity of the vibration theory, including its most recent variants. For example, shifting Raman frequencies of the ketone acetophenone by replacing its hydrogen molecules with the heavy isotope deuterium, a process called deuteration, does not alter its smell (Keller and Vosshall, 2004). One study employing the human olfactory musk-recognizing receptor, OR5AN1, found equivalent receptor activation for non-deuterated and deuterated musk ligands (Block, Jang, Matsunami, Sekharan, et al., 2015). They similarly found that receptor responses to deuterated ligands for the mouse thio receptor MOR244-3, as well as other selected human and mouse olfactory receptor proteins, do not differ from the responses of the original cognate ligands. Analogous findings employing deuterated and nondeuterated versions of p-cymene, 1-octanol, 1-undecanol, and octanol have been reported by Na, Liu, Nguyen, and Ryan (2018), although a small number of olfactory neurons (0.81% of 23, 812) did differentiate between the two versions. The authors suggested that this likely reflected subtle non-receptor processes (e.g. differences in hydrophobicity of the molecules).

As noted by Vosshall (2015) and others (e.g. Bockaert and Pin, 1999), the overwhelming evidence today is that vertebrate olfactory receptors are members of the GPCR superfamily of receptors, none of which in non-olfactory systems have been associated with vibrational mechanisms in their responses to ligands. These findings and theoretical concerns regarding electron transfer at the olfactory receptor level have led to the consensus view that receptor-centric vibrational theory is implausible. Nonetheless, there are advocates for combining elements of shape-based chemical theories, such as ones described in the next section, with elements of vibration-based theories to explain perceptual anomalies difficult to explain with either theory alone (Brookes et al., 2012).

### Theories based on chemical structure of odorants

Attempts to map chemical structures to odor qualities have a long history, including studies seeking to identify “primary odors” such as suggested by Rimmel in the 1860’s (for reviews, see von Skramlik, 1926; Moncrieff, 1967; Harper et al., 1968; Moskowitz and Warren, 1981; Ohloff, 1986; Beets, 2012). The earliest studies were observational and limited in scope, initially focusing on organic elements of the Periodic Table. Thus, two decades after the anatomic identification of the olfactory epithelium by Schultze (1862), it was noticed that the closely related elements of sulfur, selenium, and tellurium have similar odors, as do the halogens chlorine, bromine, and iodine (Ramsay, 1882; Haycraft, 1887). Ozone (O_3_) was found to have an odor, whereas oxygen was not. However, it was acknowledged that oxygen could have a smell that is not perceived because of adaptation to Earth’s oxygen-rich environment. In 1933, P. M. Niccolini concluded that odor quality is largely influenced by odorant volatility, solubility in nasal mucus, and oxidizability (Niccolini, 1933). In 1934, he noted that odorous elements are ones that fall above a diagonal line drawn from the upper left to the lower right of the periodic table—elements that have high volatility and oxygen affinity (Niccolini, 1934). He divided such “olfacto-positive” elements into those with and without an odor. He understood that some non-odorous olfacto-positive elements could, however, become odorous when combined with non-odorous elements. Others, such as barium and germanium, could acquire an odor when combined with one of the odorous elements, namely arsenic, bromine, chlorine, fluorine, iodine, or phosphorus (McCord and Witheridge, 1949).

Given awareness that odorants are commonly hydrocarbons or hydrocarbon derivatives, most studies have focused on the odor of organic chemicals. Among early observations relating such chemicals to odor were those of Jacques Passy, who concluded that the relative strength of alcohols increased as their molecular weight increased (Passy, 1892), an observation mirrored by modern threshold data (Cometto-Muniz and Cain, 1990). In 1906, Gertrud Woker found that most saturated compounds, i.e. compounds whose bonding sites are filled, are odorless and that odor increases as the degree of saturation decreases (Woker, 1906), a phenomenon also reported by other early workers (e.g. Marchand, 1915; Delange 1922). Marchand observed that strong odorants are ones that commonly form double covalent bonds, i.e. shared electrons by two atoms in their outermost shells, as occurs in aldehydes, ketones, and acids. In 1917, Eugene Louis Backman noted that the middle members of homologous series that possess both water and lipid solubility typically have the strongest smells within that series (Backman, 1917). Moncrieff (1944) summarized structure:activity findings up to that time, enumerating 62 general principles that still apply today. Examples include: 1. Lactones have fragrant ester-like odors. 2. Ketones generally have pleasant odors. 3. The architectural type of a molecule is the main factor in determining its odor. 4. An oxygen linkage is frequently associated with a pleasant odor, e.g. esters, lactones, nitrates. 5. Some substances change their odor upon dilution, e.g. indole and amines.

In light of the knowledge that three basic pigments can produce all colors and the widespread acceptance of the Young-Helmholtz theory of color vision articulated in 1875, a number of investigators followed Rimmel’s lead to find odor “primaries” from which all other odors can be analogously derived. In 1922, S. Ohma introduced the concept of cross-adaptation in efforts to identify underlying receptor classes (Ohma, 1922). Cross-adaptation is the degree to which an odorant, when continuously smelled, can reduce the sensitivity to another odorant. Conceptually, odorants that cross-adapt are employing the same receptors. However, Ohma’s efforts were limited in scope and only produced three main odor subclasses: almond, citral, and camphor/spice. Ohma’s approach was later taken up by Jacques Le Magnen during World War II, who evaluated a larger number of odorants, but he discontinued the work after finding a lack of cross-adaptation reciprocity, i.e. equivalent degrees of adaptation between the pairs of odorants he tested (Le Magnen, 2001). Others have also noted the general lack of such reciprocity (e.g. Berglund, Berglund, Engen, and Lindvall, 1971; Köster, 1971; Pierce, Wysocki, Aronov, Webb, and Boden, 1996; Stevens and O’Connell, 1996).

**Fig. 2.**
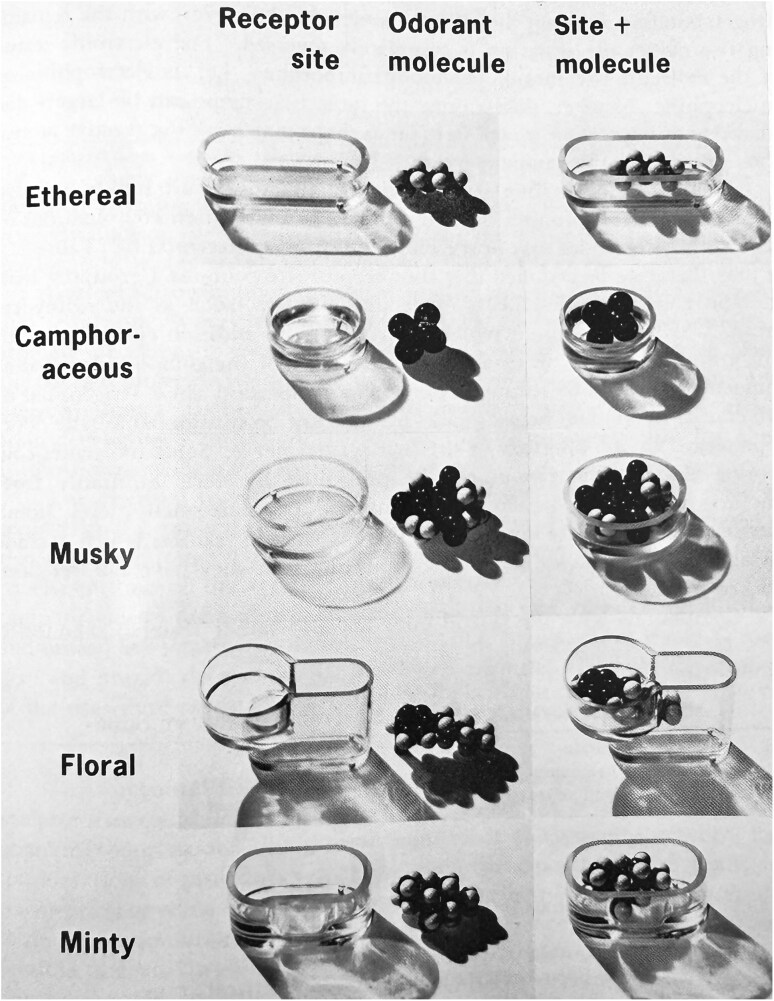
Clear plastic models of the five primary odor receptor sites proposed by J. E. Amoore (1964). The receptor site models were based on molecular sizes and shapes. Copyright 1964 The New York Academy of Sciences.

The most sophisticated and influential 20th century approach for explicating odor primaries was John Ernest Amoore’s “Stereochemical Theory of Olfaction” (Amoore, 1952). This “lock and key” theory was influenced by the Nobel Laureate Linus Pauling, in whose lab Amoore worked for a year (1959). In 1946, Pauling stated, “Even the senses of taste and odor are based upon molecular configuration rather than upon ordinary chemical properties – a molecule which has the same shape as a camphor molecule will smell like camphor even though it may be quite unrelated to camphor chemically” (Pauling, 1946) (p. 1065). Accordingly, Amoore’s initial classification system was based on the size and shape of hundreds of odorant chemicals and the names most frequently assigned to them (Amoore, 1962b). Beginning with odorants with relatively rigid chemical structures, he identified common molecular characteristics for a given smell quality. Using this and other information, he initially concluded that there were seven primary odor classes, namely ethereal, camphoraceous, musky, floral, minty, pungent, and putrid (Amoore, 1962a). Aside from the latter two categories, which depended upon electronic molecular properties, the size of these rigid molecules was the most important common chemical denominator for an odor quality, although shape also played a role. He employed scale diagrams, 3-dimensional models of molecules having different odors, and other methods to define the seven hypothetical receptor sites to which representatives of primary odor classes would fit. As an example, he indicated, “For the rather egg-shaped, camphoraceous-smelling compounds, the complementary receptor site must resemble an oval basin in shape. The smallest basin which could fit all of the rigid molecules with a camphoraceous odor would be about 9 Å long, 7½ Å wide, and 4 Å deep.” (p. 51) (Amoore, 1970). He then hypothesized that molecules that did not receive unitary names were “complex odors” which resulted from concurrent stimulation of two or more of the primary odor receptor sites. He developed standards for each of the seven classes to which the similarity of any odor could be compared. Clear plastic models of the five primary odor receptor sites involving shape, along with the fit of exemplar odorants, are shown in Fig. 2.

In his attempt to identify the primary odors, Amoore subsequently employed threshold tests and the concept of specific anosmia, explicated earlier by Guillot (1948). Persons with an otherwise normal ability to smell who can’t smell a given compound, or who have markedly elevated thresholds to that compound compared to most persons tested, were considered to have a “specific anosmia” (Amoore, 1977). Such “smell blind” persons were assumed to be the olfactory analog of color-blind persons who lack one or more of the three types of cones responsible for the normal perception of color. As a result of these studies, he amended his original thinking and concluded that there were many primary odors and classifications far beyond the original seven he had proposed.

Subsequent to the pioneering work of Amoore, dozens of 20th century studies have applied such statistical procedures as factor analysis, cluster analysis, and MDS to odor quality data in attempts to identify common associations among them (for reviews, see Yoshida, 1975; Wise, Olsson, and Cain, 2000; Chastrette, 2002; Kaeppler and Mueller, 2013). In the late 1950s, F. Nowell Jones applied factor analysis to olfactory thresholds obtained for 30 odorants (Jones, 1957). No relationship to odor quality was apparent and the thresholds were positively correlated with one another, with large individual differences in threshold values being present. Based on these data, Jones suggested that “there are probably many varieties of receptor” (p. 232), in accord with Amoore’s ultimate conclusions.

In the 1960s and 1970s, a number of investigators employed MDS in attempts to better define odor qualities and assess their physicochemical underpinnings (e.g. Gregson and Mitchell, 1974; Schiffman, 1974; Woskow, 1964; Wright and Michels, 1964; Masaaki Yoshida, 1964a, 1964b, 1972). For example, Susan Schiffman subjected a data set of 50 odorants to MDS and found that the odorants could be represented in a two-dimensional space, accounting for 91% of the variance (Schiffman, 1974). Similar findings were noted by her in a smaller data set using a different MDS analysis. In both cases, the stimuli roughly fell into two spatial groupings, pleasant and unpleasant. She then subjected each of these two groupings to separate MDS analyses and again found that two-dimensional solutions accounted for most of the variance, leading her to conclude that “… a two dimensional space adequately describes the relationships among a wide range of olfactory stimuli.” (p. 114).

When the odor qualities of the stimuli in the perceptual spaces were defined by well-established descriptors, Schiffman concluded that, overall, “the use of adjectives alone to order olfactory stimuli (a procedure employed previously by many classifiers) cannot provide a stable, clear, and unequivocal ordering of olfactory quality.” (p. 115). When molecular formulas of the olfactory stimuli were examined, with an emphasis on the size and shape of the molecules, she concluded, “Although there are certainly some trends in the spaces, especially with regard to cyclic molecules, it would be impossible to conclude from even this limited sample of chemicals that the stereochemical properties alone determine olfactory quality” (p. 115). Schiffman then proposed weighting a series of physicochemical variables to obtain a model that could, in aggregate, explain the spatial arrangements among the stimuli. This algorithm was designed to “… maximize the configurational similarity of the psychologically determined space with a space generated by physicochemical parameters” (p. 117). The correlation between 39 psychological MDS quality scaling differences and a weighted combination of their molecular weights, i.e. number of double bonds, functional groups, cyclic structures, and Raman shift intensities, was + 0.76.

A number of 21^st^ Century investigators have employed large data sets to develop models in which odorants and physicochemical parameters can be combined in multidimensional space. Among these models are those of Keller et al. (2017), Khan et al. (2007), Koulakov et al. (2011), Lee et al. (2023), Mainland et al. (2014), Ravia et al. (2020), Snitz et al. (2013) and Zarzo and Stanton (2009). As described below, however, no model, despite its sophistication, has accounted for the vast majority of perceived odor qualities.

### Challenges in relating chemical structures to odor qualities

Chemical theories have many challenges to overcome, including some of the same as vibration theories (e.g. odorants with identical functional groups and nearly identical shapes can smell differently). Importantly, many odors are based upon the combination of dozens, even hundreds, of volatile compounds synthesized into a unitary whole for which the individual components cannot be perceptually identified beyond a small number (Laing, 1987; Laing and Francis, 1989). Indeed, mixtures comprised of large numbers of diverse odorants tend to smell alike, even if they share no molecular components (Weiss et al. 2012) . In other words, olfaction is largely a synthetic sense, not an analytical one. Complexity is the rule. For example, the odor of the cultivated variety of Giant Cavendish bananas is made up of 31 odor-active volatiles from a total of 146 (Pino and Febles, 2013), although more than 1000 banana varieties exist, which can differ in key components. Twenty to 30 volatiles from around 60 are significant contributors to the perceived aroma of espresso coffee. However, this dramatically changes with the type of coffee and brewing methods (Angeloni et al., 2021).

Unlike vision, simple additive principles of stimulus combinations do not exist (Gerkin and Castro, 2015). Indeed, even monotonicity in threshold responses to odorants is often lacking, reflecting reversals or “notches” in the psychometric function relating sensitivity to odorant concentration (Cameron and Doty, 2025). Moreover, chemical interactions within the olfactory mucus cannot be ruled out, as well as both agonist and antagonist responses at each receptor from molecules within the same odorant’s mixture (Oka, Omura, Kataoka, and Touhara, 2004; Thomas-Danguin et al., 2014; de March et al., 2020; Xu et al., 2020; Xu, Zou, and Firestein, 2023). The olfactory receptor cell population also replenishes itself at intervals ranging from 30 to 60 days, requiring chronically active generative processes (Mackay-Sim, St.John, and Schwob, 2015). Small soluble odorant-binding proteins within the olfactory mucus likely facilitate movement of some odorants, e.g., hydrophobic ones, to their cognate receptors or, alternatively, remove odorants to enhance recovery of the sensitivity of the involved receptor cells (Pevsner and Snyder, 1990). The vast chemical literature on odorant structure:activity up until the late 1960’s was summarized by (Harper et al., 1968) in two sentences: “Although much information on the relation between specific areas of odour and chemical structure have been elucidated, little can be generalized to the whole field. It is still not possible to say why certain molecules are odorous when often very similar molecules with very similar vapour pressures are not” (p. 61–62).

Reliable relationships between odor quality and chemical structures, when present, appear to be limited to circumscribed classes of chemicals or “local spaces” (Jraissati and Deroy, 2021). Structure-odor relationships have been explored in great detail within the fragrance industry largely for economic reasons to develop synthetic molecules with the same odor as expensive natural products. Most success within such circumscribed odorant classes has occurred for odorants with rigid structures that can be described by one or two descriptors (e.g. ambergris, bitter almond, musk, and sandalwood) (Rossiter, 1996). Interestingly, different odors of optical isomers (molecules that are mirror images of one another) depend upon the isomer’s molecular rigidity or flexibility (Brookes, Horsfield, and Stoneham, 2009). Those that smell differently generally have rigid molecular structures, whereas those that smell the same have flexible molecular structures. Odor intensity, which is less complicated than odor quality, appears to fare best when such parameters as molecular weight, polarity, hydrophobicity, and degree of unsaturation are characterized (Edwards and Jurs, 1989), although such factors are also associated with odor quality (Mayhew et al., 2022). Odor intensity of some compounds is directly related to water solubility (Cain, 1969) and the amount of summated neural activity within the olfactory mucosa, reflecting the number of receptor cells that are activated (Doty, Kreiss, and Frye, 1990). On the basis of an extensive review of this topic, Moncrieff (1967) concluded, “There is no simple and consistent relationship between odour and <molecular> constitution. *What has to be realized now is that the dependence of odour quality on molecular configuration is only half the story and that the other half is to be found in the receptors and brain of the person or animal who is doing the smelling*” (emphasis mine) (p. 485). This same argument has been applied to attempts to identify putative pheromones in mammals (Doty, 2010).

Following up on Moncrieff’s comments, the complexity of this whole process is becoming more appreciated. As mentioned earlier, we now know, in humans, that ~400 different types of olfactory receptor cells are embedded within the 6 to 10 million receptor cells (Gilad and Lancet, 2003). Each receptor cell harbors receptor proteins of the same type (Monahan and Lomvardas, 2015). Each receptor protein can respond to more than one odorant (Malnic et al., 1999; Kajiya et al., 2001; Firestein, 2004) and can recognize multiple physicochemical features of molecules (e.g. functional groups, molecular size, charge distributions). The combination of firing of subsets of ~400 different classes of receptor cells—termed the combinatorial code—ultimately provides the first level of information critical for the central nervous system to activate circuits that ultimately provide a conscious perception of an odor (Lledo, Gheusi, and Vincent, 2005). However, spatial representations of the combinatorial code appear to break down in neural representations beyond the olfactory bulb and it is unclear how the central nervous system subsequently deals with this information (for reviews, see Doty, 2015; Gottfried, 2015; Qu, Kahnt, Cole, and Gottfried, 2016; Cleland and Linster, 2019; Rolls, 2019).

Neurobiologists can isolate individual olfactory receptors, develop cell lines, and determine the responses of the receptors to odorants using electrophysiological and other means (e.g. calcium imaging) (Peterlin, Firestein, and Rogers, 2014). When an inclusive set of agonists is identified after screening hundreds of odorant chemicals via such bioassays, the receptor is considered “deorphanized.” However, less than 20% of the ~400 human GPCRs have been deorphanized. One reason has been the difficulty in developing systems where olfactory receptor genes can be inserted in order to express large numbers of the receptor protein for subsequent study (termed cell-based heterologous expression systems). For unknown reasons, few mammalian cell lines provide an acceptable environment for such expression and their transport to the plasma membrane. In some cases, olfactory receptors get trapped within intracellular membranes, negating their ability to interact with an external odorant (Gimelbrant, Stoss, Landers, and McClintock, 1999; Lu, Echeverri, and Moyer, 2003; McClintock and Sammeta, 2003; [Bibr CIT0148][Bibr CIT0160]). While assay systems are available that incorporate the olfactory receptor neuron itself, such assays are very time-consuming. Moreover, different findings can occur across different types of assay systems, and even within a given system, considerable variability in the responses of a receptor can occur (e.g. Bozza, Feinstein, Zheng, and Mombaerts, 2002; Grosmaitre, Vassalli, Mombaerts, Shepherd, and Ma, 2006). In an insightful review of the unpredictability of odors from molecular structures, Charles Sell (Sell, 2006) concluded that “… even when the structure of an olfactory receptor is known it is far from certain that one could predict how well any novel potential ligand would bind to it” (p. 6260).

In light of the aforementioned complexities, it is perhaps not surprising that relatively recent attempts to employ advanced computational methods, including machine learning, to correlate human olfactory percepts with physicochemical properties have not fared well (Barwich, 2022). This is in spite of the employment of hundreds of odorant descriptors and thousands of physicochemical parameters in many of the algorithms. The lack of success, typified by weak to modest associations between aggregate models of such parameters and human perceptual responses (e.g. Lee et al., 2023), likely reflects not only the unreliability of indeterminant odor classifications and the potential numbers of such classes but also Moncrieff’s point that the receptors and the brain cannot be left out of models attempting to associate chemical structures with psychological percepts. As pointed out by Markus Meister, there is an obvious disconnect between the dimensionality of receptor space and perceptual space (Meister 2015): “… the dimensionality of receptor space is determined by molecular principles involving the number of ligands of interest, their relevant concentrations, energetics of ligand binding, and the design limitations of protein structures. The dimensionality of perceptual space, on the other hand, is governed by behavioral and ecological constraints: the nature of olfactory cues in the environment, the kinds of decisions the animal makes based on odorants, and the need to associate new odors with unusual events." (p. 9)

Ann-Sophie Barwich and Elisabeth A. Lloyd innumerate numerous limitations of modern structure activity studies such as that employed by Keller et al. (2017), reiterating points made above and throughout this review (Barwich and Lloyd, 2022). This includes variability and lack of specificity of the involved odorant nomenclatures (e.g. “garlic” is specific, “chemical” is ambiguous, and “spices” is very broad) and that semantic descriptors are not sound representations of perceptions. They argue that the assumption that structure-odor-rules are innate is wrong, neglecting the roles of experience, context, and culture. Moreover, they point out that such theories fail to consider biological variables associated with perceptual responses to stimuli, including the fact that single receptors are responsive to numerous odorants. Importantly, they note the disparity between the linearity employed in current machine learning structure-odor-rules and the non-linear receptor responses of odorants, although other models have since emerged that are less constrained (Ameta et al., 2025).

Leslie B. Vosshall provided a state-of-the-art summary in 2015, which remains true today: “After centuries of conjecture on how a molecule leads to a smell percept, we still lack a convincing framework to predict the smell of a molecule from its chemical structure” (p. 6526) (Vosshall, 2015). William S. Cain provided an earlier overview of the challenges of establishing associations between physicochemical odorant parameters and the perception of odor qualities as deduced by perfumers (Cain, 1988):

Will the search for structural commonality in various similar smelling compounds ever help to understand olfaction? Perhaps yes and perhaps no. The search arises from certain aspects of categorical perception that may actually mislead. In the case with a family of chemicals with similar smells, structure-activity researchers commonly tend to view each material as comprising a fundamental odor quality with accompanying overtones. Such descriptions foster the illusion of, for example, an amber-smelling core to a molecule and certain accompanying non-amber molecular features. Any anticipated isomorphism between phenomenological properties and molecular properties is gratuitous (p. 440).

## Are “odor objects” the ultimate cognitive classification system?

As can be gleaned from this review, odorants are complex chemical stimuli commonly comprised of dozens and sometimes hundreds of components, which confound attempts to associate their odor with chemical structures in a straightforward manner. Given this complexity, one is led to the heuristic hypothesis that odors are cognitive concepts dependent upon higher-order CNS processes that integrate initial spatial and temporal patterns of peripheral olfactory receptor activity with brain mechanisms involved in semantics, learning, and memory. The role of learning in dictating an odorant’s meaning is in accord with William James’s view that “every perception is an acquired perception” (James, 1890) (p. 78). An extensive review of the role of learning in determining the meaning of odors was provided by Wilson and Stevenson in a book entitled “Learning to Smell” (Wilson and Stevenson, 2006). In 2003, these authors noted that “early analytically processing of odors is inaccessible at the behavioral level and that all odors are initially encoded as ‘objects’ in the piriform cortex. Moreover, we suggest that odor perception is wholly dependent on the integrity of this memory system and that its loss severely impairs normal perception” (Wilson and Stevenson, 2003) (p. 243). The concept of odor objects was described by Jay Gottfied (2015):

The term “odor object” refers to an olfactory source (such as a rose bush) or an olfactory event (such as the aroma of a rose) that is presented to the senses. This is not different from conventional notions of objects in the visual domain, such as visual sources (rose bush) and visual events (the light reflecting off a rose bush). It is the distinctive physical and ecological form of sensory objects that shapes nervous system function (Gottfried, 2009, 2010). The fact that most smells naturally encountered in the environment are complex mixtures of dozens or even hundreds of different odorant molecules means that the olfactory system needs mechanisms to weave these odor elements into perceptual wholes. … Anatomical, physiological, and computational studies in animal models suggest that odor object information may take the form of distributed ensemble patterns of activity in the piriform cortex (Haberly, 1985; Hasselmo et al.1990; Illig and Haberly, 2003), but how odor objects are represented in the human brain is unclear. (p. 292)

According to Wilson and Stevenson (2006), the fundamental premise of this theory is that “… odor objects are learned through experience. Odorants and odorant features that co-occur are synthesized through plasticity within central circuits to form single perceptual outcomes that are resistant to background interference, intensity fluctuations, or partial degradation. Learned odor objects may include multimodal components and recognition of familiar objects can be sharpened by context, attention and expectation.” (p. 2) In effect, this theory accepts the complexity of an odorant mixture as a “configuration” which the brain must translate.

Heuristically, the odor object concept appears to have merit, although conceiving odor objects as analogous to visual objects has been questioned. Thus, some authors have argued that such parallelism is fallaceous, as it fails to address the major differences between the two sensory systems. For example, Barwich (2019) points out that, in color vision, all three classes of photoreceptors respond to the same stimulus property, wavelength. In contrast, the nearly 400 olfactory receptors respond in a non-linear non-additive fashion to ~5000 different chemical parameters. Hence, feature extraction is not homogenous in olfaction and “ultimately, odor qualities are determined by the constitution and coding of the receptors, not the chemical topology of the stimulus.” (p. 5) Barwich also points out that, in contrast to vision, where most persons harbor the same set of visual cones, marked genetic variations occur in olfactory receptors, resulting in large differences among individuals in their olfactory perceptual repertoire (Keller et al., 2017; Trimmer et al., 2019). Numerous mutations within the olfactory genome are known, some of which may reflect specific anosmias or hyposmias such as those noted by Amoore. One example is a mutation near the olfactory receptor gene OR6A2. Carriers of this mutation experience the smell of cilantro (coriander) as pungent and soapy, rather than fruity or green (Eriksson et al., 2012). Barwich also argues that, unlike visual objects, odors are often ambiguous, lacking distinctive features (also see Olofsson and Gottfried, 2015). Moreover, in contrast to visual objects, adaptation occurs rapidly to odors and their meaning is context dependent, being influenced by cross-modal cues, verbal descriptors, selective attention, expectation, and other factors noted earlier in this review (e.g. age, odorant concentration, context). Visual objects retain their structure and do not adapt, even when viewed from different perspectives, and are not as strongly influenced by such extraneous factors.

Regardless of the degree to which odor objects parallel visual objects, if one accepts them as key elements of a classification system, a simple array of coincident odorant classification is not possible, since millions of odor objects and subtle variations thereof are conceivable. Even a given odor object, such as chocolate, can have multiple learned variants based not only on smell, but on vision and touch (e.g. milk chocolate, dark chocolate, white chocolate, unsweetened chocolate, soft chocolate, etc.). While this approach appears to provide a logical empirical-based explanation of odor percepts useful in exploring brain processes, such percepts go beyond the olfactory system, per se, and continue the dilemma, and more likely the reality, that odors cannot be classified into interpretable universal vector-related categories.

## Data Availability

No new data were generated or analysed in support of this review.

## Notes

This research did not receive any specific grant from funding agencies in the public, commercial, or not-for-profit sectors. This paper is based, in part, on a keynote address to the symposium, “Universal Chemosensory Testing: Challenges and Opportunities,” held in Philadelphia, PA, on November 7, 2023.
